# Frequency of toxin genes and antibiotic resistance pattern of *Clostridioides difficile* isolates in diarrheal samples among hospitalized patients in Hamadan, Iran 

**Published:** 2021

**Authors:** Leili Shokoohizadeh, Fatemeh Alvandi, Abbas Yadegar, Masoumeh Azimirad, Seyed Hamid Hashemi, Mohammad Yousef Alikhani

**Affiliations:** 1 *Department of Microbiology, Faculty of Medicine, Hamadan University of Medical Sciences, Hamadan, Iran*; 2 *Foodborne and Waterborne Diseases Research Center, Research Institute for Gastroenterology and Liver Diseases, Shahid Beheshti University of Medical Sciences, Tehran, Iran*; 3 *Brucellosis Research Center, Hamadan University of Medical Sciences, Hamadan, Iran*

**Keywords:** Clostridioides difficile, TcdA, TcdB, Binary toxin, Antibiotic resistance

## Abstract

**Aim:**

This study was designed to investigate the prevalence of *Clostridioides difficile*, its toxin-producing genes, and antibiotic resistance patterns in diarrheal samples from hospitalized patients in Hamadan, Iran.

**Background:**

Today, concerns over *Clostridioides difficile* infection (CDI) have significantly increased due to reduced susceptibility to antibiotics used for CDI treatment. Toxins produced by *C. difficile* strains are associated with disease severity and outcome.

**Methods:**

In this cross-sectional study, a total of 130 diarrheal samples of patients admitted to different wards of three hospitals in Hamadan from November 2018 to September 2019 were collected. *C. difficile* isolates were identified by culture on CCFA and PCR (Polymerase chain reaction). The presence of toxin-encoding genes (*tcdA* and *tcdB*) and binary toxin genes (*cdtA* and *cdtB*) was analyzed by PCR. Resistance of the isolates to metronidazole, vancomycin and clindamycin antibiotics was determined using agar dilution method.

**Results:**

Out of 130 diarrheal samples from hospitalized patients, 16 (12.3%) *C. difficile* isolates were obtained. PCR results were positive for two toxin-producing genes, *tcd*A and *tcdB*, in all (100%) *C. difficile* isolates, and the binary toxin genes *cdt*A and* cdtB* were detected in 6 (37.5%) and 8 (50%) isolates, respectively. The results of antibiotic susceptibility testing showed resistance to metronidazole, vancomycin, and clindamycin in 3 (18.7%), 3 (18.7%), and 2 (12.5%) isolates, respectively, and all isolates were resistant to rifampicin.

**Conclusion:**

The results of this study showed toxigenic *C. difficile* with *tcdA*^+^*/tcdB*^+^ profile is a major cause of nosocomial diarrhea in Hamadan, and clinical laboratories should routinely perform *C. difficile* diagnostic testing on diarrheal specimens of hospitalized patients. Resistance to conventional antibiotic therapy against *C. difficile* should be considered as a warning to prevent irrational administration of antibiotics.

## Introduction

*Clostridioides difficile* is a Gram-positive, obligate anaerobic bacterium that forms spores and is considered an important human pathogen. Attention to this bacterium has increased since it was described in 1978 as the main cause of antibiotic-induced diarrhea and the development of almost all cases of pseudomembranous colitis and toxic megacolon (1). *C. difficile* was initially thought to be a hospital-acquired bacterium, but there are some reports of *C. difficile* infection (CDI) among people outside hospitals or people who had not taken antibiotics. However, increasing prevalence in the community compared to the hospital is reported to be 1300 times lower, which may be due to lower use of antibiotics in the community (2).

*C. difficile* causes colitis and diarrhea by producing two exotoxins: toxin A (enterotoxin) and toxin B (cytotoxin). In human studies, the level of toxins in the stool is related to the severity of the disease. Toxin A causes inflammation, which leads to the secretion of intestinal fluids and mucosal damage. Toxin B is approximately 10 times more involved in colonic mucosal damage than toxin A, suggesting that toxin B may be more critical than toxin A in the pathogenesis of *C. difficile* colitis. A few strains of *C. difficile* can produce another toxin called binary toxin, which consists of a cell binding component and an enzymatic component that shows an action-specific ADP ribosyltransferase activity leading to disorganization of the cytoskeleton (3-5).

Various risk factors affect the prevalence of *C. difficile* nosocomial infections, which include antibiotics, advanced age, hospitalization, debilitating diseases such as cancer or treatment with immunosuppressive drugs, abdominal surgery, chemotherapy, and prolonged stay in a healthcare setting. Two major roles for antibiotics in the pathogenesis of *C. difficile* have been described. In first, antibiotics destroy the normal intestinal flora and provide conditions for *C. difficile* to multiply and produce toxins. Second, the rapid growth of *C. difficile* resistance to clindamycin and fluoroquinolones seems to play an important role in increasing the prevalence and pathogenicity of this microbe (6).

Antibiotics that play a major role in predisposing the host to *C. difficile*-associated diarrhea include fluoroquinolones, clindamycin, a range of penicillins and cephalosporins. Any antibiotic, even metronidazole and vancomycin, used to treat *C. difficile* can cause antibiotic-dependent colitis. Although an increased resistance to metronidazole has been observed in clinical isolates of *C. difficile*, it is still the most effective agent in the treatment of infections caused by this bacterium (5, 7-9).

Currently, there is insufficient information about the prevalence of *C. difficile* in the west of Iran. Thus, the aim of the present study was to determine the frequency of *C. difficile*, its toxin genes, and resistance to metronidazole, vancomycin and clindamycin among isolates obtained from fecal samples of patients with diarrhea who were admitted to hospitals in Hamadan. 

## Methods

**Patients and** ***C. difficile*** **clinical isolates**

In this cross-sectional study, a total of 130 fecal specimens were collected from hospitalized patients with diarrhea in the hospitals of Hamadan from November 2018 to September 2019. This study was approved by the Ethics Committee of Hamadan University of Medical Sciences (IR.UMSHA. REC.1397.510). Diarrhea was defined as the passage of more than two loose or watery stools during a 24 h period or fewer hours (10). Fecal samples from patients were collected in a specific stool container and then immediately transferred to the microbiology laboratory in Hamadan University of Medical Sciences. Stool samples were treated as previously described (11, 12). To isolate *C. difficile, *the treated suspensions were cultured on cycloserine-cefoxitin fructose agar (CCFA; Mast Co, UK) supplemented with 5% fresh sheep blood and incubated anaerobically for 48 h at 37 °C using an anaerobic jar (MART Microbiology B.V. the Netherlands). Identification of the isolates was performed based on Gram staining, odor and colony characteristics on CCFA plates. For molecular confirmation of *C. difficile* isolates, the *cdd3*, as a housekeeping gene (*C. difficile* downstream 3), was targeted by PCR (Polymerase chain reaction) using specific primers as described previously by Cohen et al. (13). Samples confirmed as *C. difficile *were stored in cooked meat broth (Merck, Germany) at 4 °C, and were subjected to further molecular identification. A questionnaire containing demographic data and time of hospitalization, age and sex of patient was completed for patients.

**DNA extraction and PCR**

Genomic DNA was extracted from freshly grown colonies using the boiling method. All *C. difficile* isolates were subjected for determination of toxin genes. The detection of toxin A gene (*tcdA*), toxin B gene (*tcdB*), and binary toxin genes (*cdtA* and *cdtB*) was performed by PCR described by Cohen et al. and Terhes et al., using specific primers (Metabion, Germany) shown in Table 1 (13, 14). The PCR reactions for the detection of *tcdA* and *tcdB* genes were done in a total volume of 25 μL. The reaction mixture contained 10 µl master mix (Amplicon, Denmark), 0.5 μM of each primer, 1 μL DNA template, and 10 μL distilled water. The PCR reactions consisted of an initial denaturation step at 95 °C for 5 min, followed by 30 cycles of 60 sec at 95 °C, annealing for 45 sec at 51 °C (for *tcdA*), 50 °C (for *tcdB*), 53 °C (for *cdtA *and *cdtB*), and extension for 50 sec at 72 °C. A final extension step was performed at 72 °C for 5 min. The PCR products were separated by electrophoresis in 1.2% agarose gels.

**Table 1:** Primer sequences and fragment lengths used for amplification of *cdd3*, *tcdA*,* tcdB*, *cdtA,* and *cdtB* genes

Primer (gene)	Nucleotide sequence	Fragment Length (bp)	References
*cdd3*	F: 5´ TCC AAT ATA ATA AAT TAG CAT TCC A 3´ R: 5´ GGC TAT TAC ACG TAA TCC AGA TA 3´	622	
*tcdA*	F: 5´ ATG ATA AGG CAA CTT CAG TGG 3´ R: 5´ TAA GTT CCT CCT GCT CCA TCA A 3´	624	
*tcdB*	F: 5´ GAG CTG CTT CAA TTG GAG AGA 3´ R: 5´ GTA ACC TAC TTT CAT AAC ACC AG 3´	412	
*cdtA*	F: 5ʹ TGA ACC TGG AAA AGG TGA TG 3ʹ R: 5ʹ AGG ATT ATT TAC TGG ACC ATT TG 3´	375	
*cdtB*	F: 5ʹ CTTAATGCAAGTAAATACTGAG 3ʹ R: 5ʹ AACGGATCTCTTGCTTCAGTC 3ʹ	512	

**Antimicrobial susceptibility testing**

Antimicrobial susceptibility of *C. difficile* isolates to metronidazole and clindamycin and was determined using the breakpoints defined by the Clinical and Laboratory Standards Institute (document M11-A8) and to vancomycin by the European Committee for Antimicrobial Susceptibility Testing (EUCAST) criteria (15). The MIC breakpoints for vancomycin and rifampicin were used as previously described (16). The minimum inhibitory concentration (MIC) of these antibiotics (Sigma-Aldrich, St. Louis, Mo) was determined by the agar dilution method. The range of MIC value used for antimicrobial agents was 0.5 to 256 μg/ml. Media with different concentrations of each antibiotic were prepared by adding defined amounts of each antibiotic to cooled Brucella agar medium supplemented with hemin (5 μg/ml), vitamin K1 (10 μg/ml), and 5% sheep blood (5). The turbidity of each bacterial suspension was adjusted equivalent to a no.1 McFarland standard, and 20 μl of each suspension was inoculated on Brucella agar plates containing different concentrations of each antibiotic. Inoculated plates without antibiotics served as control.

**Statistical analysis**

The data was analyzed using SPSS software, version 21 for Windows (SPSS Inc., Chicago, IL, USA). A *p-*value <0.05 was considered statistically significant. 

## Results

Out of 130 collected stool specimens, 16 (12.36%) were *C. difficile* culture positive and confirmed with the *cdd3* gene PCR. Of 130 stool samples, 70 (53.8%) were collected from women and 60 (46.1%) were from men. For men, 7 cases (11.6%) and for women, 9 cases (12.8%) were positive for the presence of *C. difficile*. The results indicate that *C. difficile* is more common in diarrhea specimens isolated from women; however, there was no significant difference according to the gender of patients (*p* = 0.38). Based on the length of hospital stay of the patients, 50% of *C. difficile*-positive patients were hospitalized for more than 7 days (longest time), the shortest time (12.5%) was at 1 to 3 days, and 37.5% of *C. difficile*-positive patients were hospitalized for 3 to 7 days. In this study, the age range of patients who showed *C. difficile* infection ranged from 28 to 89 years; 8 (50%) of the positive cases were in the age range of 50 to 70 years old, and 6 (37.5% ) and 2 (12.5%) positive cases were in 70-90 and 28-50 age ranges, respectively. Most (87.5%) *C. difficile-*positive samples were isolated from patients in the internal wards of the hospitals; only 2 (12.5%) positive samples were isolated from patients in the intensive care unit (ICU).

**Figure 1:**
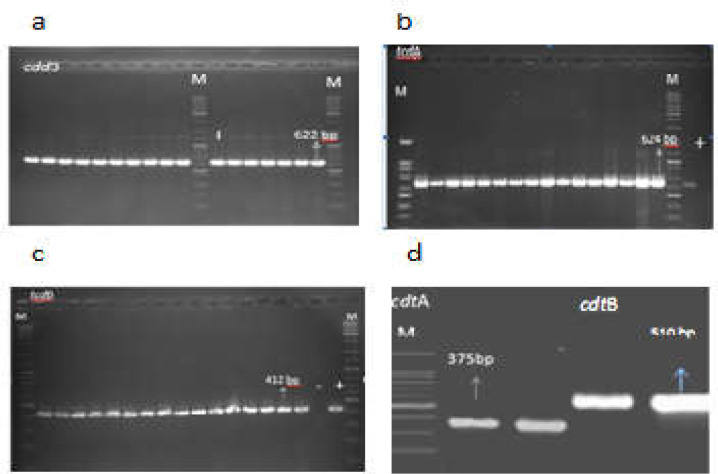
Gel electrophoresis of PCR products for *C. difficile* identification and toxin encoding genes in isolates from hospitalized patients in Hamadan. (a) *cdd3*; (b)* tcdA*; (c) *tcdB*; (d) *cdtA *and* cdtB *genes. Lane M, 100 bp DNA size marker

All *C. difficile* isolates (100%) carried both *tcdA* and *tcdB* genes, showed a *tcdA*^+^*/tcdB*^+^ profile, and were considered as toxigenic strains. The *cdtA* and *cdtB* genes were detected in 6 (37.5%) and 8 (50%) isolates, respectively. Two isolates were negative for both *cdtA* and *cdtB* genes. Simultaneous detection of binary toxin genes did not occur in any of the isolates. Therefore, *tcdA/tcdB/cdtA* and *tcdA/tcdB/cdtB* profiles were detected in 6 (37.5%) and 8 (50%) of the isolates, respectively (Figure 1). The details of *C. difficile* isolates obtained in this study are shown in Table 2.

**Table 2:** Characteristics of *C. difficile* isolates from hospitalized patients in Hamadan

Isolates	Patient sex	Ward	*tcdA*	*tcdB*	*cdtA*	*cdtB*
1	M	Int	+	+	-	+
2	M	Int	+	+	-	+
3	F	Int	+	+	-	+
4	M	Int	+	+	+	-
5	F	Int	+	+	+	-
6	F	Int	+	+	-	+
7	M	Int	+	+	-	+
8	F	ICU	+	+	-	+
9	F	Int	+	+	+	-
10	F	Int	+	+	+	-
11	M	ICU	+	+	-	+
12	F	Int	+	+	+	-
13	M	Int	+	+	-	+
14	F	Int	+	+	+	-
15	F	Int	+	+	-	-
16	F	Int	+	+	-	-

**M**, male; **F**, female; **Int**, internal ward; **ICU**, intensive care unit

**Table 3:** Interpretive criteria of the MIC values for *C. difficile* isolates

Antibiotic agent	(μg/ml)			No. of isolates with MIC of (µg/ml)										Susceptibility profile		
	S	I	R	0.5 1 2 4 8 16 32 64 128 256										S (%)	I (%)	R (%)
Metronidazole^*^	≤8	16	≥32	12	1	-	-	-	-	-	-	3	-	81.3	-	18.7
Vancomycin^§^	≤2	4	≥8	12	-	-	1	3	-		-	-	-	75	6.3	18.7
Rifampicin^§^	-	-	≥4	-	-	-	-	16	-	-	-	-	-	-	-	100
Clindamycin^*^	≤2	4	≥8	1	-	-	13	-	2	-	-	-	-	6.3	81.2	12.5

Breakpoints were defined as susceptible (S), intermediately resistant (I), or resistant (R) with reference to CLSI (*) or published data (§)

According to antimicrobial susceptibility testing by agar dilution, resistance to metronidazole (MIC ≥32 μg/ml), clindamycin (MIC ≥8 μg/ml), and vancomycin (MIC ≥8 μg/ml) was observed in 3 (18.7%), 2 (12.5%), and 3 (18.7%) of the isolates, respectively. All isolates were resistant to rifampicin (MIC ≥4 μg/ml). Reduced susceptibility to vancomycin and clindamycin was observed in one (6.2 %) and 13 (81.2 %) of isolates, respectively, which was interpreted as intermediate phenotype (MIC = 4 μg/ml). The interpretive criteria of the MIC values for *C. difficile* isolates are shown in Table 3.

## Discussion

This research is the first study of the prevalence of CDI in diarrheal samples of patients admitted to hospitals in Hamadan. The prevalence of CDI was detected to be 12% in this study. It was established that the incidence rate of CDI is significantly higher among hospitalized people than among those who acquired it from the community (17, 18). The rate of CDI in different countries has been reported as being from zero to 36% (19-22). Currently, there is limited data about the molecular epidemiology of CDI in Africa, Asia, and Latin America (23, 24). It is difficult to obtain accurate epidemiological information about the prevalence of CDI in developing countries, because their diagnosis is based on immunoassay (EIA) methods rather than culture (25-27). Limited laboratory capacity, inefficient infection management and control systems affect the accurate reporting of CDI prevalence in developing countries (28, 29). In recent years, several studies were performed on anaerobic bacteria due to the provision of facilities and optimizing of anaerobic culture and isolation methods. In a study by Borren et al., which included 51 studies from throughout Asia, the rate of CDI was 14.8% among all patients with diarrhea and showed a higher prevalence in East Asia (19.5%) compared with South Asia (10.5%) or the Middle East (11.1%) (30). Pooled prevalence of CDI in Persian Gulf countries ranged from 2.8% to 21.7% (31). In Saudi Arabia, the prevalence of CDI varied from 17% to 20% in suspected diarrheal samples (32, 33). The current findings also showed a lower prevalence of CDI compared to some studies from Saudi Arabia; however, the prevalence of CDI in hospitals in Kuwait and Qatar is lower than the current results (34, 35). Moreover, the current findings showed a prevalence rate of CDI similar to that in South and East Asia, such as India (10.9%), China (14%), and South-Korea (14.3%) (36-38). According to studies conducted from 2010 to 2020 in Iran, different prevalence rates of *C. difficile* ranging from 4.7% to 39% have been reported (5, 10, 12, 39-41). The prevalence of CDI in hospitals in Hamadan is lower than that in Tehran, Isfahan, and Kerman (10, 12, 42). This variation in the prevalence of CDI in different studies might be due to differences in geographical distribution, infection control policies, diagnostic methods, or the studied population (30).

The role of toxins A and B in the pathogenicity of *C. difficile* is well studied. In the current study, all isolates were toxigenic and showed a *tcdA*^+^/*tcdB*^+^/CDT^-^ profile, because none of the isolates contained binary toxins simultaneously, but 6 (37.5%) and 8 (50%) of the isolates contained *cdtA* and *cdtB* genes, respectively. Due to the importance of the TcdB toxin in bacterial pathogenesis, the presence of the *tcdB* gene in all isolates is important and indicates that we are facing high virulence strains in the hospitals of Hamadan. Multiple studies have reported the *tcdA*^+^/*tcdB*^+^/CDT^-^ profile as a main toxin profile. In a study done by Shoaei et al., 11.5% of clinical samples from inpatients in hospitals of Isfahan were toxigenic and showed a *tcdA*^+^/*tcdB*^+^/CDT^-^ profile; only one (2.2%) isolate harbored all toxin-associated genes with *tcdA*^+^, *tcdB*^+^, *cdtA*^+^, and *cdtB*^+^ profiles (10).

Rezazadeh et al. reported different results in diarrheal samples from ICU patients in Kerman (43). In their study, the frequency of CDI was 41%, and *tcdA*^+^/*tcdB*^+^/CDT^-^ profiles were detected in 20% of isolates; only one isolate with the *tcdA*^+^/*tcdB*^+^/CDT^+^ profile was detected. The dominance of the *tcdA*^+^/*tcdB*^+^/CDT^-^ profile has also been reported by Heidari et al., Goudarzi et al., and Azimirad et al. from hospitals in Tehran and Shiraz (5, 12, 39). Moreover, the *tcdA*^+^/*tcdB*^+^/CDT^-^ profile was reported in 18.8%, 92%, 8%, and 71% of *C. difficile* isolates from Japan, Czech Republic, Argentina, and China, respectively (4, 44, 45). Consistent with the current results, the presence of one of the two binary genes in the clinical strains of *C. difficile* has been reported in most studies from Asian countries such as Japan, Korea, China, and Thailand (10, 46). In European countries, however, the prevalence of binary toxins has been reported in 4% to 12% of *C. difficile* isolates, and these strains have been associated with higher mortality and CDI recurrence (47, 48).

*C. difficile* is the main infectious cause of antibiotic-associated diarrhea (AAD), and metronidazole and vancomycin remain as the first-line drugs in the treatment of CDI. One of the main goals of the current study was to determine the prevalence of resistance to metronidazole, vancomycin and clindamycin. Resistance to metronidazole, clindamycin and vancomycin was observed in this study. *C. difficile* isolates showed a geographically dispersed antibiotic resistance pattern due to the use of different standards and susceptibility testing methods. In European countries and the United States, E-test has been the most common method for testing antimicrobial susceptibility of *C. difficile* (45). Most results are reported according to CLSI criteria (49). Resistance to metronidazole has been reported in various parts of the world, being first reported in 2011-2012 (50). According to the results of various studies, the rate of resistance to metronidazole was reported to be from zero to 18.3% until 2018. The results also showed that resistance to metronidazole has decreased by about 0.8%, and this dramatic decrease observed in some countries may be due to the choice of test for antibiotic susceptibility testing (49). In recent years, vancomycin and metronidazole resistance has been reported in Iran and other countries. According to the results of previous studies, the proportions of vancomycin non-susceptibility varied from 0 to 87.7% in BI/NAP1/027 isolates (51-54). This data indicates an increase in vancomycin resistance over time. High rates of vancomycin resistance have been reported in the United States and then in Asia (7, 55). Vancomycin resistance averaged 0.4% before 2012 and reached 4% afterward, that shows a significant difference (7, 56). One of the reasons for the increase in vancomycin resistance is the extended use of vancomycin in US hospitals, after which resistance to vancomycin significantly increased worldwide (49). In 2013, the first report of vancomycin resistance was reported to be 1% among *C. difficile* isolates in Tehran (39). In the current study, resistance to vancomycin was detected in three isolates (18.7%), and a decreasing trend in the susceptibility of vancomycin (intermediate phenotype) was detected in 6.2% of the isolates. Three isolates were also found to be resistant to metronidazole. Resistance to clindamycin was found to be lower than resistance to vancomycin and metronidazole in this study. In various studies from European, Asian, and North American countries, resistance to clindamycin has been reported to be from 8.3% to 100% (46, 57, 58). There are different reports on resistance to vancomycin, metronidazole, and clindamycin. Based on recent studies in Iran by Shoaei et al. from Isfahan and Heidari et al. from Shiraz, the antimicrobial susceptibility determination by E-test showed that all toxicogenic *C. difficile* isolates were sensitive to vancomycin and metronidazole (5, 10), while Baghani et al. reported that 30% of *C. difficile* strains isolated from Tehran hospitals were resistant to vancomycin (59). Baghani et al. also reported that 81.5% of the isolates were resistant to metronidazole, which showed a high rate of resistance to this antibiotic and raised great concern about the treatment of patients with CDI (59). In a study conducted by Mohammadbeigi et al., all *C. difficile* isolates were susceptible to vancomycin, and 8.16% and 72.1% of toxigenic isolates were resistant to metronidazole and clindamycin, respectively, in Kerman hospitals (60). Some studies from China showed susceptibility to vancomycin in 100% and resistance to metronidazole in 15.6-35.3% of *C. difficile* isolates (61, 62). In Europe, resistance to therapeutic antibiotics of choice in the case of CDI, like metronidazole and vancomycin, showed low resistance rates of 0.1% and 2.3%, respectively (62, 63).

To conclude, a relatively high frequency of *C. difficile* was detected in diarrheal samples collected from two hospitals in Hamadan. The data also indicated that the *tcdA*^+^/*tcdB*^+^/CDT^-^ toxigenic pattern was predominant among *C. difficile* isolates, and clinical laboratories should routinely perform *C. difficile* diagnostic tests on diarrheal specimens of hospitalized patients in this area. 

There were some limitations in our study, such as a small sample size, the lack of molecular typing information of the isolates by ribotyping method, and insufficient equipment for anaerobic culture of bacteria. The rate of resistance to conventional antibiotic therapy against *C. difficile* was alarming in Hamadan. Thus, further studies are needed to monitor the rate and pattern of antibiotic resistance of *C. difficile* isolates in this region.
